# Evaluation of Treatment Response in Chronic Hepatitis C Patients Receiving Sofosbuvir/Velpatasvir/Voxilaprevir: A Multicenter Real-World Experience from Türkiye

**DOI:** 10.3390/v17070931

**Published:** 2025-06-30

**Authors:** Umut Devrim Binay, Faruk Karakeçili, Bilgehan Aygen, Ayşin Kılınç Toker, İlhami Çelik, Neşe Demirtürk, Tuğçe Şimşek Bozok, Leyla Dursun, Fethiye Akgül, Güle Çınar, Özgür Günal, Ali Asan, Eyüp Arslan, Fatma Yılmaz Karadağ, Orçun Barkay, İrem Akdemir, Funda Şimşek, Emine Türkoğlu Yılmaz, Zeynep Ravza Eğilmez, Süda Tekin, The Viral Hepatitis Study Group of the Turkish Society of Clinical Microbiology and Infectious Diseases (KLİMİK)

**Affiliations:** 1 Department of Infectious Diseases and Clinical Microbiology, Faculty of Medicine, Erzincan Binali Yıldırım University, 24100 Erzincan, Türkiye; drfarukkarakecili@hotmail.com (F.K.); o.barkay1985@gmail.com (O.B.); 2 Department of Infectious Diseases and Clinical Microbiology, Faculty of Medicine, Erciyes University, 38030 Kayseri, Türkiye; baygen@erciyes.edu.tr (B.A.); dursunleyla96@gmail.com (L.D.); zeynepravzayuksel@gmail.com (Z.R.E.); 3 Department of Infectious Diseases and Clinical Microbiology, Kayseri City Hospital, University of Health Sciences, 38080 Kayseri, Türkiye; draysin@gmail.com (A.K.T.); ilhamicelik@hotmail.com (İ.Ç.); 4 Department of Infectious Diseases and Clinical Microbiology, Faculty of Medicine, Afyonkarahisar Health Sciences University, 03030 Afyonkarahisar, Türkiye; nesedemirturk@yahoo.com; 5 Department of Infectious Diseases and Clinical Microbiology, Faculty of Medicine, Mersin University, 33343 Mersin, Türkiye; tugce_0103@hotmail.com; 6 Department of Infectious Diseases and Clinical Microbiology, Batman Training and Research Hospital, 72070 Batman, Türkiye; dr.fethiyeakgul@gmail.com; 7 Department of Infectious Diseases and Clinical Microbiology, Faculty of Medicine, Ankara University, 06230 Ankara, Türkiye; gbinjune@gmail.com (G.Ç.); iremakd@yahoo.com (İ.A.); 8 Department of Infectious Diseases and Clinical Microbiology, Faculty of Medicine, Samsun University, 55080 Samsun, Türkiye; ozgurgop@yahoo.com; 9 Department of Infectious Diseases and Clinical Microbiology, Bursa Yüksek İhtisas Training and Research Hospital, University of Health Sciences, 16310 Bursa, Türkiye; draasan@yahoo.com; 10 Department of Infectious Diseases and Clinical Microbiology, Sancaktepe Şehit Prof. Dr. İlhan Varank Training and Research Hospital, University of Health Sciences, 34785 İstanbul, Türkiye; dreyuparslan@hotmail.com (E.A.); dr_fatma@hotmail.com (F.Y.K.); 11 Department of Infectious Diseases and Clinical Microbiology, Prof. Dr. Cemil Taşçıoğlu City Hospital, University of Health Sciences, 34384 İstanbul, Türkiye; fundasimsek67@gmail.com; 12 Department of Infectious Diseases and Clinical Microbiology, Faculty of Medicine, Tokat Gaziosmanpaşa University, 60030 Tokat, Türkiye; eminee43@hotmail.com; 13 Department of Infectious Diseases and Clinical Microbiology, Acıbadem Ataşehir Hospital, 34642 İstanbul, Türkiye; suda.tekin@gmail.com

**Keywords:** sofosbuvir/velpatasvir/voxilaprevir, hepatitis C treatment, real-world data

## Abstract

The combination of sofosbuvir/velpatasvir/voxilaprevir (SOF/VEL/VOX) is recommended as a salvage therapy for treatment-experienced chronic hepatitis C (CHC) patients. However, it is used in our country for treatment-naïve and treatment-experienced patients. This study aims to present real-world data from Türkiye on CHC patients treated with SOF/VEL/VOX. The present study was conducted by the Viral Hepatitis Study Group of the Turkish Society of Clinical Microbiology and Infectious Diseases (KLİMİK). It was a multicenter, retrospective, observational study. The data were collected from patients receiving SOF/VEL/VOX therapy at 12 medical centers in Türkiye between 1 June 2022 and 31 December 2024. The patients had received the treatment for 8 to 12 weeks. Of the 139 patients enrolled, 63.3% (*n* = 88) were male, with a mean age of 54.4 years. Most patients were non-cirrhotic (94.2%, *n* = 131) and treatment-naïve (92%, *n* = 128); 49.6% (*n* = 69) were infected with genotype 1b. Early virologic response (EVR) could be assessed in 126 patients, with an EVR rate of 82.5% (*n* = 104). End-of-treatment data were available for 113 patients, all achieving an end-of-treatment response. Among the 80 patients for whom week-12 post-treatment data were available, 97.5% sustained virologic response at week 12 (SVR12). Significant improvements were observed in AST, ALT, and platelet levels, along with reductions in APRI and FIB-4 scores (*p* = 0.001).” No serious adverse events leading to treatment discontinuation were reported. Mild adverse events included pruritus (2.1%, *n* = 3), fatigue (2.1%, *n* = 3), and nausea (1.4%, *n* = 2). The SOF/VEL/VOX combination is a highly effective and well-tolerated treatment option in treatment-naïve CHC patients, achieving an SVR12 rate of 97.5%.

## 1. Introduction

Hepatitis C virus (HCV) infection is a communicable disease that primarily affects the liver but can also involve extrahepatic systems. Acute HCV infection is often asymptomatic; however, approximately 70% of cases progress to chronic hepatitis C (CHC). CHC remains a leading cause of liver cirrhosis and hepatocellular carcinoma (HCC), accounting for approximately 242,000 deaths globally in 2022, according to the World Health Organization (WHO). It is estimated that around 50 million people are currently living with HCV worldwide, with nearly 1 million new infections occurring annually [1].

Pan-genotypic direct-acting antivirals (DAAs) have revolutionized CHC treatment, offering sustained virological response (SVR) rates exceeding 95%. Without a prophylactic vaccine for HCV, timely diagnosis and treatment are critical components of the WHO’s 2030 global hepatitis elimination goals [2].

The fixed-dose combination of sofosbuvir (an NS5B polymerase inhibitor), velpatasvir (an NS5A inhibitor), and voxilaprevir (an NS3/4A protease inhibitor) [SOF/VEL/VOX] was approved by the United States Food and Drug Administration (FDA) on 18 July 2017, for use in patients with HCV genotypes 1 through 6, including those with compensated or decompensated cirrhosis [3].

Although the SOF/VEL/VOX combination is recommended by international guidelines such as the European Association for the Study of the Liver (EASL) [4] and the American Association for the Study of Liver Diseases (AASLD) [5] for use in treatment-experienced patients, it has been included in the reimbursement system in Türkiye since June 2022 [6] for both treatment-naïve and treatment-experienced patients. In Türkiye, the regimens used in treatment-experienced patients are limited. CHC treatment is covered free of charge according to the conditions determined by the social security institution. For treatment-experienced patients, 12 weeks of SOF/VEL/VOX combination or 16 weeks of GLE/PIB + Ribavirin combination is recommended for non-cirrhotic or compensated cirrhotic patients; 24 weeks of SOF/LED + Ribavirin is recommended for decompensated cirrhotic patients [6,7]. In contrast, the combination of sofosbuvir/velpatasvir (SOF/VEL) has not been made available in Türkiye. Therefore, despite being recommended by both national and international guidelines as a salvage therapy, the SOF/VEL/VOX combination is widely used in treatment-naïve patients in Türkiye. The efficacy and safety of the SOF/VEL/VOX combination in real-world settings among treatment-experienced patients have been demonstrated in various studies [8,9]. Although Phase 3 clinical trials such as POLARIS-2 and POLARIS-3 [10] have shown that the SOF/VEL/VOX combination is effective and safe in treatment-naïve patients, the present study aims to evaluate the real-world efficacy and safety of the SOF/VEL/VOX regimen specifically in treatment-naïve patients.

## 2. Materials and Methods

The study was designed as a multicenter, retrospective, observational cohort study coordinated by the Viral Hepatitis Study Group of the Turkish Society of Clinical Microbiology and Infectious Diseases (KLİMİK). Ethical approval was obtained from the Erzincan Binali Yildirim University Clinical Research Ethics Committee (Decision no.: 2024-01/3, Date: 11 January 2024), and all necessary permissions were obtained from the participating centers. The study was conducted in accordance with the Declaration of Helsinki. As a retrospective study, informed consent was not a prerequisite.

The study population comprised patients over the age of 18 who had been diagnosed with CHC and who had received SOF/VEL/VOX (400/100/100 mg) therapy. Data from 139 patients treated between 1 June 2022 and 31 December 2024 at 12 medical centers across Türkiye were evaluated.

At the time of initial presentation to infectious diseases and clinical microbiology outpatient clinics, patients routinely underwent complete blood count, biochemical, and molecular testing (including HCV genotyping and quantitative HCV RNA assessment). These parameters were also monitored during treatment follow-up. The following data were retrieved from the patients’ medical records: demographic characteristics, laboratory values, history of CHC treatment, status of hepatic fibrosis, changes in concomitant medications due to drug–drug interactions, and adverse events.

The duration of treatment, which was eight or twelve weeks, was determined by prior treatment experience and the presence or absence of cirrhosis. An HCV RNA level that remains below the lower limit of quantification after the initial month of treatment is designated as an early virologic response (EVR). The presence of undetectable HCV RNA after treatment was selected as an end-of-treatment response (ETR). In contrast, undetectable HCV RNA at week 12 following the completion of therapy was characterized as a sustained virologic response (SVR12).

Non-invasive indices were utilized to assess liver fibrosis, including the aspartate aminotransferase (AST) to platelet ratio index (APRI) and fibrosis-4 (FIB-4) score. The APRI score was used to assess liver status and cirrhosis. The standard formula (APRI = (AST [U/L]/upper limit of normal AST [U/L]) ×100/platelet count [10^9^/L]) was used to determine the APRI score. The center where the study was conducted calculated the APRI score, considering the last AST and platelet count before treatment. An APRI score of ≤0.3 was classified as no cirrhosis or fibrosis, >0.3 and ≤0.5 as no cirrhosis and fibrosis possible, >0.5 and ≤1.5 as cirrhosis and fibrosis possible, >1.5 and ≤2 as cirrhosis possible and fibrosis, and >2 as probable cirrhosis [11]. The FIB-4 score is calculated using the following formula: a score below 1.45 suggests the absence of fibrosis, while a score greater than 3.25 indicates the presence of advanced fibrosis [12]. FIB-4 = [age (years) x AST(U/L)/platelet level (10^9^/L) x alanine aminotransferase (ALT) (U/L)½].

HCV RNA levels were quantified by using the Roche COBAS Ampliprep/COBAS TaqMan HCV Test, v2.0 (Roche Molecular Systems, Inc., Branchburg, NJ, USA), which has a lower limit of quantification (LLOQ) of 15 IU/mL.

### Statistical Analysis

Statistical analyses were performed using SPSS (Statistical Package for the Social Sciences) version 27 (IBM corp., Armonk, NY, USA). Quantitative variables are presented as mean, standard deviation, median, minimum, and maximum values, while qualitative variables are summarized using frequency and percentage. The Shapiro–Wilk test and box plot graphs assessed the normality of data distribution. For variables not showing normal distribution with three or more repeated measures, within-group comparisons were performed using the Friedman test with Wilcoxon signed-rank test corrections. Results were considered statistically significant at a *p*-value < 0.05 with a 95% confidence interval.

## 3. Results

The study encompassed a total of 139 patients from 12 medical centers. The end-of-first-month data were available for 126 patients, the end-of-treatment data for 113 patients, and the week 12 post-treatment data (SVR12) for 80 patients. Of the total cohort, 63.3% (*n* = 88) were male, and the mean age was 54.4 years (see Table 1).

Of the patients, 94.2% (*n* = 131) were non-cirrhotic, and 92% (*n* = 128) were treatment-naïve. Among the 11 treatment-experienced patients, previous regimens included glecaprevir/pibrentasvir (GLE/PIB) (*n* = 4), interferon-based therapies (*n* = 3), sofosbuvir-based regimens (*n* = 3), and paritaprevir/ritonavir/ombitasvir/dasabuvir with ribavirin (*n* = 1).

Coinfections were identified in a small subset: 9.3% (*n* = 13) had resolved Hepatitis B Virus (HBV) infection, 2.9% (*n* = 4) had active HBV coinfection, and none had Human Immunodeficiency Virus (HIV) coinfection.

Comorbid conditions were reported in 43.9% (*n* = 61) of patients. The most common comorbidities in patients were essential hypertension (29.5%), type 2 diabetes mellitus (8%), and coronary artery disease (5%). Two patients, both involving antihypertensive agents, had drug modifications due to drug–drug interactions. Liver biopsy was performed in only two patients (1.5%).

The baseline HCV RNA levels ranged from 210 to 90,990,042 IU/mL, with a mean of 3,974,462.36 ± 9,339,575.64 IU/mL. Genotype data indicated that 49.6% (*n* = 69) of the subjects were infected with genotype 1b, while genotype information was not available in 22.3% (*n* = 31) (see Table 2).

Table 3 presents the distribution of baseline laboratory results of the patients.

All 113 patients (100%) who returned for the end-of-treatment evaluation achieved an end-of-treatment response. In the per-protocol efficacy analysis, SVR12 was achieved in 78 patients (97.5%), while relapse was observed in 2 patients (2.5%); 42.4% of the patients were lost during the SVR12 follow-up and in the intention to treat analysis, SVR12 was achieved at a rate of 56.1%. All treatment-experienced patients with available end-of-treatment data (*n* = 5) achieved SVR12. The two patients who experienced relapse were non-cirrhotic and infected with non-genotype 3 HCV (Table 4).

**Figure 1:**
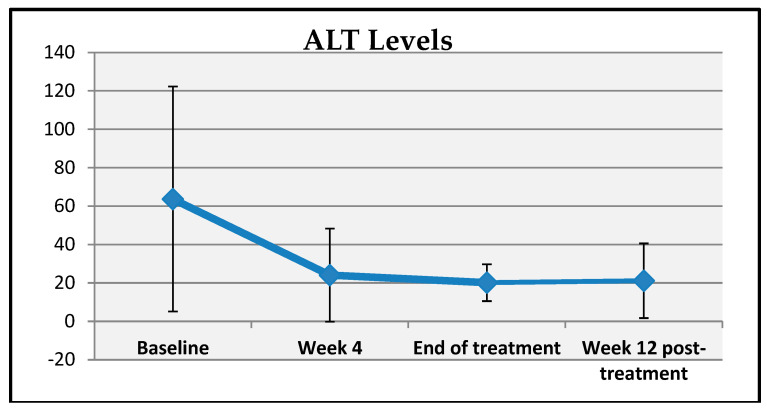
Changes in ALT measurements over time.

A statistically significant change was observed in patients’ ALT levels at baseline, week 4, end of treatment, and week 12 post-treatment (*p* = 0.001; *p* < 0.01). A comparative analysis of the mean changes in ALT levels at weeks 4, 8, and 12 post-treatment revealed a statistically significant decrease (*p* = 0.001; *p* < 0.01) (Table 5, Figure 1). The mean reduction in ALT levels at week 4, the end of treatment, was 38.96 ± 51.13 units, while the mean decrease at week 8 and week 12 post-treatment was 41.17 ± 58.47 and 36.81 ± 46.62 units, respectively.

**Figure 2:**
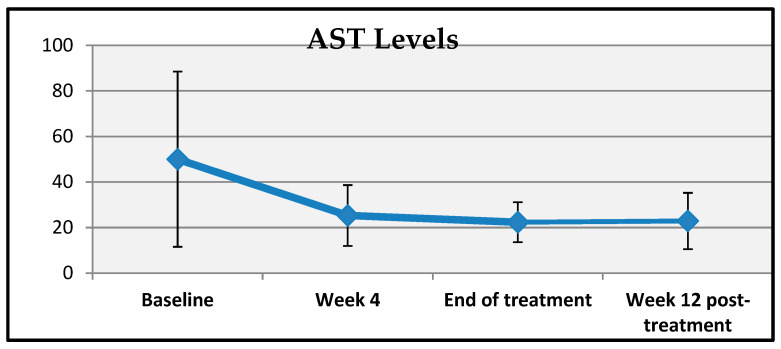
Changes in AST measurements over time.

A statistically significant change was observed in patients’ AST levels at baseline, week 4, end of treatment, and week 12 post-treatment (*p* = 0.001; *p* < 0.01). A comparative analysis of the mean changes in AST levels at weeks 4, 8, and 12 post-treatments revealed a statistically significant decrease (*p* = 0.001; *p* < 0.01) (Table 6, Figure 2). The mean reduction in AST levels at week 4, the end of treatment, was 23.26 ± 37.27 units, while the mean decrease at week 8 and week 12 post-treatment was 26.76 ± 38.70 and 24.77 ± 35.26 units, respectively.

**Figure 3:**
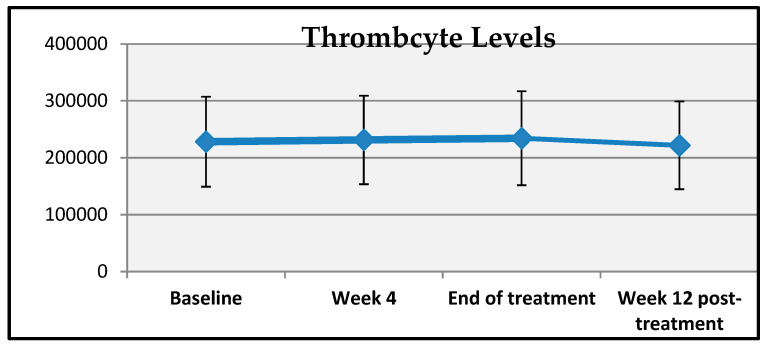
Changes in thrombocyte measurements over time.

A statistically significant change was observed in patients’ platelet counts at baseline, week 4, end of treatment, and week 12 post-treatment (*p* = 0.001; *p* < 0.01). Compared to baseline, the mean increase in platelet count at week 12 post-treatment was 7.509,3 ± 62,480 units, which was statistically significant (*p* = 0.001; *p* < 0.01) (Table 7, Figure 3).

**Figure 4:**
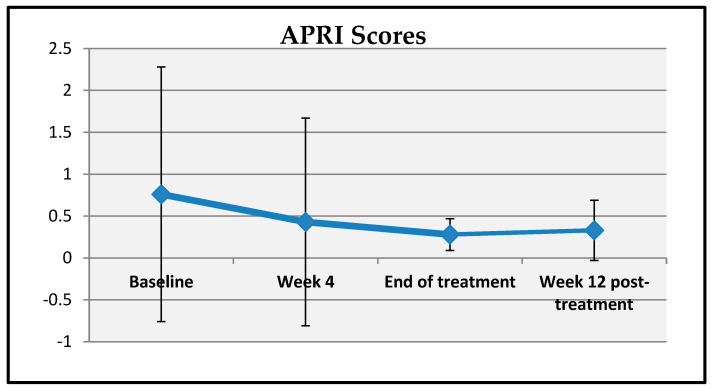
Changes in APRI scores over time.

A statistically significant change was observed in patients’ APRI scores at baseline, week 4, end of treatment, and week 12 post-treatment. Compared to baseline, the mean decreases in APRI scores at week 4, end of treatment, and week 12 post-treatment were 0.33 ± 1.95, 0.47 ± 1.69, and 0.35 ± 0.62 units, respectively, which were statistically significant (*p* = 0.001; *p* < 0.01) (Table 8, Figure 4).

**Figure 5:**
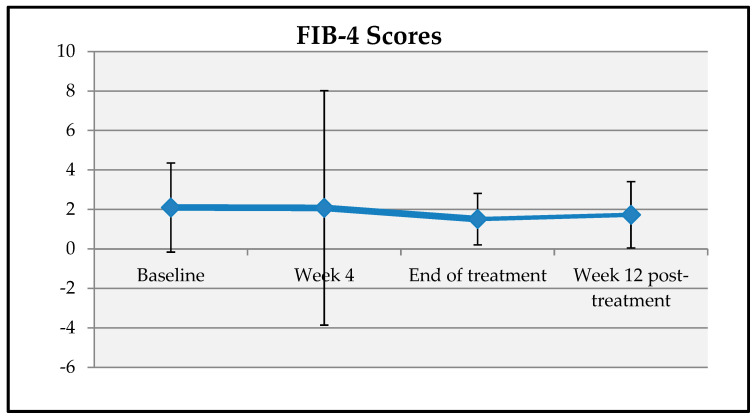
Changes in FIB-4 scores over time.

A statistically significant change was observed in patients’ FIB-4 scores at baseline, end of treatment, and week 12 post-treatment. Compared to baseline, the mean decreases in FIB-4 scores at the end of treatment and week 12 post-treatment were 0.69 ± 1.90 and 0.57 ± 2.51 units, respectively, which were statistically significant (*p* = 0.001; *p* < 0.01) (Table 9, Figure 5).

No serious adverse events requiring treatment discontinuation were observed in any patient. Pruritus and fatigue were reported in three patients each, while nausea was observed in two patients. Prophylactic HBV treatment (entecavir or tenofovir alafenamide fumarate) was initiated in nine patients before therapy. In one patient, Entecavir was started during the first month of treatment due to HBV reactivation.

## 4. Discussion

This study is one of the most substantial investigations of real-world data on treatment-naïve CHC patients treated with the SOF/VEL/VOX combination. Although SOF/VEL/VOX is primarily recommended as a rescue therapy, it is also used in treatment-naïve patients in countries like ours where the SOF/VEL combination is not available. Consequently, disseminating real-world data concerning SOF/VEL/VOX utilization in treatment-naïve patients is paramount.

Real-world SOF/VEL/VOX data are mostly reported from treatment-experienced patient groups. In a systematic review by Devan et al., which analyzed data from 24 studies including 2877 treatment-experienced patients, the SVR12 rate in the per-protocol (PP) population was 95%. Lower treatment success was observed in patients infected with genotype 3, those with prior SOF/VEL exposure, cirrhotic patients, and patients with HCC. Baseline resistance-associated substitutions (RASs) were shown not to affect SVR12 [13]. Similarly, a meta-analysis by Xie et al., including 15 studies and 1796 treatment-experienced patients, reported a PP SVR12 rate of 96%, with lower SVR12 rates among genotype 3-infected, cirrhotic, and SOF/VEL-experienced patients [14]. Papaluca et al. reported an SVR12 rate of 90% in a difficult-to-treat cohort predominantly composed of NS5A inhibitor-experienced genotype 3 and cirrhotic patients [15]. A study from Spain analyzing 142 patients previously treated with SOF/VEL or GLE/PIB showed an SVR12 rate of 88.6% [8]. Prospective data from Canada also showed similar outcomes [16].

A study in Türkiye by Çakırca et al. reported a 100% sustained virologic response (SVR12) rate. This study included 41 treatment-naïve patients, and the SVR12 results were available for 23 patients [17]. In the present study, the SVR12 data from 75 treatment-naïve and 5 treatment-experienced patients were available 12 weeks after treatment. The SVR12 rate was achieved in all treatment-experienced patients and 97.3% of treatment-naïve patients. All eight cirrhotic patients achieved the SVR12, and all four genotype 3-infected patients with available data also achieved SVR12. The findings from our cohort demonstrate the remarkable efficacy of the SOF/VEL/VOX combination in treatment-naïve patients.

No serious adverse events leading to treatment discontinuation were observed in our study. Adverse events were limited to pruritus (2.1%), fatigue (2.1%), and nausea (1.4%). In contrast, Liu et al. from Taiwan reported hyperbilirubinemia in 15% of their cohort [18], whereas Da et al. reported no adverse events [19]. Degasperi et al., in a study including 179 treatment-experienced patients, reported hyperbilirubinemia and fatigue in 6% and anemia in 4% of patients [20]. A Korean study observed headache, nausea, and rash in 9.1% of patients, but no treatment discontinuations due to adverse events [9]. Rustam and Qaisar’s systematic review reported serious adverse events in 3.24% of patients, including nausea, fatigue, diarrhea, and abdominal pain as drug-related effects [21]. Another meta-analysis reported treatment discontinuation due to adverse events in 0.66% of cases [14], while a different meta-analysis found serious adverse events at 1.94%, many of which were not drug-related [13]. Most data in the literature come from treatment-experienced patients. Among treatment-naïve patients reported from Türkiye, nausea and diarrhea were observed at a rate of 2.4% (1 patient) [17]. Including 128 treatment-naïve patients in our study is therefore significant and demonstrates the safety of the SOF/VEL/VOX combination in treatment-naïve patients in real-world settings.

The primary goals of CHC treatment are to prevent the development of cirrhosis and HCC, thereby reducing HCV-related mortality and preventing viral transmission [2]. Achieving viral clearance reduces inflammation and fibrosis regression and improves liver function [4,5]. In our study, the significant improvements observed in APRI and FIB-4 scores and favorable changes in ALT, AST levels, and platelet counts compared to baseline values support these effects.

Our study has some limitations. These include the small number of cirrhotic and genotype 3-infected patients, the lack of pre-treatment RAS analysis, and the study’s retrospective nature, which may introduce bias in adverse event reporting. Additionally, 42.4% of the patients were lost during the SVR12 follow-up, which is one of the important limitations of the study. Patients may have been lost to follow-up due to the continuing COVID-19 pandemic, even though it had been under control [22], and patients not being aware that they needed to come for a follow-up visit, not feeling the need to come to the follow-up because they feel well, or being a person who inject drugs [23,24]. For these reasons, it is important to provide patients with more detailed information about the disease, the importance of SVR12 control, and not to skip their follow-up when starting antiviral treatment.

## 5. Conclusions

Our study demonstrates that the SOF/VEL/VOX combination is effective and safe in treatment-naïve CHC patients.

## Figures and Tables

**Table 1:** Distributions of descriptive features.

		*N* (%)
**Gender**	Male	88 (63.3)
	Female	51 (36.7)
Age	Mean ± SD	54.4 ± 17.81
	Median (Min−Max)	58 (18–85)
Weight (kg) (*n* = 75)	Mean ± SD	74.8 ± 10.46
	Median (Min−Max)	75 (42–97)
Height (cm) (*n* = 75)	Mean ± SD	167.81 ± 8.83
	Median (Min−Max)	170 (150–185)
BMI (kg/m^2^) (*n* = 75)	Mean ± SD	26.69 ± 4.16
	Median (Min−Max)	26.2 (16.41–37.59)

SD: standard deviation; Min: minimum; Max: maximum.

**Table 2:** Distributions of disease-related characteristics.

		*N* (%)
Cirrhosis status	Non-cirrhotic	131 (94.2)
	Compensated cirrhotic	8 (5.8)
Treatment status	Naïve Experienced	128 (92)
		11 (8)
HBV coinfection Previous HBV infection No coinfections		4 (2.9)
		13 (9.3) 122 (87.8)
Comorbidity	None	78 (56.1)
	Yes	61 (43.9)
* Change due to drug–drug interaction	None	59 (96.7)
	Yes	2 (3.3)
Liver biopsy	None	137 (98.5)
	Yes	2 (1.5)
HCV RNA (IU/ML)	Mean ± SD	3,974,462.36 ± 9,339,575.64
	Median (Min−Max)	886,553 (210−90,990,042)
Genotype/subtype	Type 1a	14 (10.1)
	Type 1b	69 (49.6)
	Type 2	8 (5.8)
	Type 3	8 (5.8)
	Type 4 Genotype not assessed	9 (6.4) 31 (22.3)

* Antihypertensive medications were modified in two patients.

**Table 3:** The distribution of baseline laboratory results.

Parameter (Unit)	Mean ± SD	Median (Min−Max)
INR	1.04 ± 0.13	1 (0.8–1.8)
PT (s)	12.05 ± 2.35	11.7 (1–23.4)
FBG (mg/dL)	103.69 ± 45.08	93 (67–433)
Cholesterol (mg/dL)	168.48 ± 38.97	172 (66–245)
Triglyceride (mg/dL)	122.99 ± 60.75	98 (29–304)
HDL cholesterol (mg/dL)	46.74 ± 14.09	44.5 (10–105)
BUN (mg/dL)	19.31 ± 10.28	17 (7–62)
Creatinine (mg/dL)	0.82 ± 0.36	0.8 (0.2–4.3)
GGT (U/L)	54.35 ± 37.66	39 (9–195)
ALP (U/L)	66.64 ± 52.07	75 (1.5–229)
LDH U/L)	214.37 ± 61.29	200 (124–511)
Uric acid (mg/dL)	4.86 ± 1.36	4.9 (1.1–8.3)
Na (mmol/L)	138.54 ± 3.1	139 (130–146)
K (mmol/L)	4.32 ± 0.56	4.4 (1.1–5.2)
Total protein (g/dL)	7.2 ± 0.67	7.2 (4.5–8.4)
Albumin (g/dL)	4.37 ± 0.42	4.4 (3.5–5.5)
Total bilirubin (mg/dL)	0.6 ± 0.32	0.5 (0.1–1.8)
Direct bilirubin (mg/dL)	0.32 ± 0.49	0.2 (0–5)
AFP (ng/mL)	5.31 ± 9.73	2.9 (0.8–81)
CRP (mg/dL)	2.8 ± 3.96	1.9 (0–29)
ALT (U/L)	63.69 ± 58.54	45 (10–400)
AST (U/L)	50.05 ± 38.49	40 (16–265)
Platelet level (/mm^3^)	228,131.78 ± 79,069.33	226,000 (27,000–411,000)
Hemoglobin (g/L)	14.24 ± 1.88	14.5 (7.7–17.9)
Absolute neutrophil count (/mm^3^)	4401.93 ± 2464.95	3940 (1020–22,400)

INR: international normalized ratio; PT: prothrombin time; FBG: fasting blood glucose; HDL: high-density lipoprotein; BUN: blood urea nitrogen; GGT: gamma glutamyl transferase; ALP: alkaline phosphatase; LDH: lactate dehydrogenase; AFP: alpha-fetoprotein; CRP: C-reactive protein; ALT: alanine transaminase; AST: aspartate transaminase; SD: standard deviation; Min: minimum; Max: maximum.

**Table 4:** Distribution of treatment results.

		*N* * (%)
EVR	Treatment-naïve Experienced	104 (82.5) 22 (17.5)
ETR	Treatment-naïve Experienced	108 (100) 5 (100)
Cirrhotic	SVR12 Relapse	8 (100) 0 (0)
Treatment-experienced	SVR12 Relapse	5 (100) 0 (0)
Treatment-naïve	SVR12 Relapse	73 (97.3) 2 (2.7)

* Data were available for 126 patients at the end of the first month of treatment, 113 patients at the end of the second month, and 80 patients at week 12 post-treatment. EVR: early virologic response; ETR: end-of-treatment response.

**Table 5:** Comparison of ALT levels at baseline, week 4, end of treatment, and week 12 post-treatment.

		ALT	^a^ *p*
Baseline (*n* = 139)	Mean ± SD	63.69 ± 58.54	0.001 **
	Median (Min−Max)	45 (10–400)	
Week 4 (*n* = 126)	Mean ± SD	24.07 ± 24.26	
	Median (Min−Max)	18 (6–213)	
End of treatment (*n* = 113)	Mean ± SD	20.16 ± 9.59	
	Median (Min−Max)	18 (7–54)	
Week 12 post-treatment (*n* = 80)	Mean ± SD	21.14 ± 19.45	
	Median (Min−Max)	16.5 (6–147)	
Change Δ		Mean ± SD	^aa^ *p*
Baseline—Week 4		−38.96 ± 51.13	0.001 **
Baseline—End of treatment		−41.17 ± 58.47	0.001 **
Baseline—Week 12 Post-treatment		−36.81 ± 46.62	0.001 **

^a^ Friedman test and ^aa^ Wilcoxon signed-rank test; ** *p* < 0.01; SD: standard deviation; Min: minimum; Max: maximum; ALT: alanine aminotransferase.

**Table 6:** Comparison of AST levels at baseline, week 4, end of treatment, and week 12 post-treatment.

		AST	^a^ *p*
Baseline (*n* = 139)	Mean ± SD	50.05 ± 38.49	0.001 **
	Median (Min−Max)	40 (16–265)	
Week 4 (*n* = 126)	Mean ± SD	25.32 ± 13.36	
	Median (Min−Max)	21 (7–82)	
End of treatment (*n* = 113)	Mean ± SD	22.38 ± 8.79	
	Median (Min−Max)	21 (7–54)	
Week 12 post-treatment (*n* = 80)	Mean ± SD	22.89 ± 12.36	
	Median (Min−Max)	20 (8–80)	
Change Δ		Mean ± SD	^aa^ *p*
Baseline—Week 4		−23.26 ± 37.27	0.001 **
Baseline—End of treatment		−26.76 ± 38.70	0.001 **
Baseline—Week 12 Post-treatment		−24.77 ± 35.26	0.001 **

^a^ Friedman test and ^aa^ Wilcoxon signed-rank test; ** *p* < 0.01; SD: standard deviation; Min: minimum; Max: maximum; AST: aspartate aminotransferase.

**Table 7:** Comparison of platelet counts at baseline, week 4, end of treatment, and week 12 post-treatment.

		Thrombocyte Count	^a^ *p*
Baseline (*n* = 139)	Mean ± SD	228,131.78 ± 79,069.33	0.024 *
	Median (Min−Max)	226,000 (27,000–411,000)	
Week 4 (*n* = 126)	Mean ± SD	231,447.2 ± 77,827.2	
	Median (Min−Max)	229,000 (10,900–460,000)	
End of treatment (*n* = 113)	Mean ± SD	234,358.89 ± 82,570.08	
	Median (Min−Max)	235,000 (196–453,000)	
Week 12 post-treatment (*n* = 80)	Mean ± SD	221,855.26 ± 77,092.97	
	Median (Min−Max)	220,500 (21,000–416,000)	
Change Δ		Mean ± SD	^aa^ *p*
Baseline—Week 4		6311.43 ± 56,348.84	1.000
Baseline—End of treatment		11,522.65 ± 59,672.85	0.096
Baseline—Week 12 Post-treatment		7509.3 ± 62,480	0.036 *

^a^ Friedman test and ^aa^ Wilcoxon signed-rank test; * *p* < 0.05; SD: standard deviation; Min: minimum; Max: maximum.

**Table 8:** Comparison of APRI scores at baseline, week 4, end of treatment, and week 12 post-treatment.

		APRI	^a^ *p*
Baseline (*n* = 139)	Mean ± SD	0.76 ± 1.52	0.001 **
	Median (Min−Max)	0.5 (0.1–16.4)	
Week 4 (*n* = 126)	Mean ± SD	0.43 ± 1.24	
	Median (Min−Max)	0.3 (0.1–13.8)	
End of Treatment (*n* = 113)	Mean ± SD	0.28 ± 0.19	
	Median (Min−Max)	0.2 (0.1–1.3)	
Week 12 post-treatment (*n* = 80)	Mean ± SD	0.33 ± 0.36	
	Median (Min−Max)	0.2 (0.1–2.4)	
Change Δ		Mean ± SD	^aa^ *p*
Baseline—Week 4		−0.33 ± 1.95	0.001 **
Baseline—End of treatment		−0.47 ± 1.69	0.001 **
Baseline—Week 12 Post-treatment		−0.35 ± 0.62	0.001 **

^a^ Friedman test and ^aa^ Wilcoxon signed-rank test; ** *p* < 0.01; APRI: AST to platelet ratio index; SD: standard deviation; Min: minimum; Max: maximum.

**Table 9:** Comparison of FIB-4 scores at baseline, week 4, end of treatment, and week 12 post-treatment.

		FIB-4	^a^ *p*
Baseline (*n* = 139)	Mean ± SD	2.10 ± 2.26	0.001 **
	Median (Min−Max)	1.4 (0.2–15.5)	
Week 4 (*n* = 126)	Mean ± SD	2.08 ± 5.94	
	Median (Min−Max)	1.2 (0.3–65.5)	
End of treatment (*n* = 113)	Mean ± SD	1.51 ± 1.30	
	Median (Min−Max)	1.2 (0.2–9.55)	
Week 12 post-treatment (*n* = 80)	Mean ± SD	1.73 ± 1.68	
	*Median (Min−Max)*	1.2 (0.4–12.6)	
Change Δ		Mean ± SD	^aa^ *p*
Baseline—Week 4		−0.05 ± 5.84	0.100
Baseline—End of treatment		−0.69 ± 1.90	0.001 **
Baseline—Week 12 Post-treatment		−0.57 ± 2.51	0.001 **

^a^ Friedman test and ^aa^ Wilcoxon signed-rank test; ** *p* < 0.01; SD: standard deviation; Min: minimum; Max: maximum; FIB-4: fibrosis-4 score.

## Data Availability

The datasets generated and/or analyzed during the current study are available from the corresponding author on reasonable request.

## Abbreviations

The following abbreviations are used in this manuscript:

AASLD	American Association for the Study of Liver Diseases
AFP	Alpha-fetoprotein
ALP	Alkaline phosphatase
ALT	Alanine transaminase
APRI	AST to platelet ratio index
AST	Aspartate transaminase
BUN	Blood urea nitrogen
CHC	Chronic hepatitis C
CRP	C-reactive protein
DAAs	Direct-acting antivirals
EASL	European Association for the Study of the Liver
EOT	End-of-treatment response
EVR	Early virologic response
FBG	Fasting blood glucose
FIB-4	Fibrosis-4 score
GGT	Gamma glutamyl transferase
HCC	Hepatocellular carcinoma
HCV	Hepatitis C virus
HDL	High-density lipoprotein
INR	International normalized ratio
LDH	Lactate dehydrogenase
PT	Prothrombin time
SOF/VEL/VOX	Sofosbuvir/Velpatasvir/Voxilaprevir
SVR	Sustained virological response
WHO	World Health Organization
